# Prevalence of hypertension and prehypertension and its associated cardioembolic risk factors; a population based cross-sectional study in Alkharj, Saudi Arabia

**DOI:** 10.1186/s12889-018-6216-9

**Published:** 2018-11-29

**Authors:** Abdurrahman Aldiab, Mamdouh M. Shubair, Jamaan M. Al-Zahrani, Khaled K. Aldossari, Sameer Al-Ghamdi, Mowafa Househ, Hira Abdul Razzak, Ashraf El-Metwally, Hoda Jradi

**Affiliations:** 1 Department of Medicine, Oncology Division, College of Medicine, King Saud University, Riyadh, Saudi Arabia; 2 School of Health Sciences, University of Northern British Columbia (UNBC), 3333 University Way, Prince George, BC V2N 4Z9 Canada; 3 Department of Family Medicine, College of Medicine, Prince Sattam Bin Abdulaziz University, Al-Kharj, Saudi Arabia; 4 King Abdullah International Medical Research Center (KAIMRC)/College of Public Health & Health Informatics, King Saud Bin Abdulaziz University For Health Sciences, Riyadh, Saudi Arabia; 5 Ministry of Health and Prevention, Dubai, United Arab Emirates; 6 College of Public Health and Health Informatics, King Saud Bin AbdulAziz University for Health Sciences, Mail Code 2350, P.O. Box 3660, Riyadh, 11481 Saudi Arabia; 7 Docent of Epidemiology, School of Health Sciences, University of Tampere, Tampere, Finland

**Keywords:** Hypertension, Prehypertension, Blood pressure, Normotensive, Hypertensive, Risk factors, Cardioembolic, Saudi Arabia

## Abstract

**Background:**

Hypertension and prehypertension pose significant public-health and clinical challenges for both economically developed and developing nations. Prevalence of these conditions are frequently underreported because of its often-silent nature. Population-based studies that explore the occurrence and correlates of these conditions are scarce in Saudi Arabia. This study aimed at estimating the prevalence and associated factors of hypertension and prehypertension on a representative sample of males and females living in Al-Kharj town in Saudi Arabia.

**Methods:**

Cross-sectional analysis was performed from January 2016 until June 2016 by recruiting a representative sample (*n* = 1019; aged 18 to 67 years) of the Al Kharj population. All participants completed a self-administered questionnaire, followed by a physical examination and blood test. Statistical analysis was carried out using SPSS version 24.0 for Windows.

**Results:**

The prevalence of prehypertension was 66.1, 48.1 and 54.9% in male, female and all subjects, respectively. The prevalence of hypertension was 6.0, 4.2 and 4.9% in male, female and all subjects, respectively. Being overweight was associated with the highest risk of hypertension (OR = 4.98 [95% C.I. = 1.98–12.52], *P* = 0.001). People who were classified as class I obese had 3.5 times the risk of hypertension compared with the non-obese group (OR = 3.49 [95% C.I. = 1.42–8.63], *P* = 0.007). Risk of pre-hypertension was significantly lower in females (OR = 0.48 [95% C.I. = 0.32–0.71]) and tends to increase with obesity status. Gender-specific analyses found that males in the lowest education attainment level had a significantly increased risk of pre-hypertension (OR = 6.56 [95% C.I. = 1.27–33.85], *P* = 0.003).

**Conclusion:**

This population-based study in Saudi Arabia shows that hypertension and prehypertension are common conditions particularly among males. Overweight and obesity was associated with both conditions. In addition, lower education attainment was a significantly associated factor among males. Future prospective studies are needed to confirm the etiological nature of such associations.

## Background

Hypertension is a treatable factor which contributes to the burden of disease globally. Hypertension is one of an insidious onset disease that damages indiscernibly the fragile capillary beds in many organs such as kidney or may cause rapid rupture of blood vessels causing hemorrhage in organs such as brain. It is a main risk factor for cardiovascular morbidity and mortality, surpassing obesity, diabetes mellitus, and smoking [1]. Hypertension is a major predictor of premature death and cardiovascular disability that poses a huge economic burden to both medical cost and human capital loss [2–4].

The prevalence of hypertension and prehypertension is significantly increased with rapid expansion, economic development, acceleration of population aging, modifications in lifestyle and traditional dietary habits. Globally, 13.5% of the total premature deaths (7.6 million), 54% of stroke, and 47% of ischemic heart disease were attributed to hypertension alone [5]. In Saudi Arabia, levels of hypertension have been reported to range from 26.1% among the 30–70 years age group in 1995–2000 [6] to 25.5% among the 15–64 years old age group in 2005 [7, 8]. According to 2010 estimates, hypertension was classified as the leading risk factor for death in Saudi Arabia [9]. The most recent report about cardiovascular risk factors among the Saudi population presented a prevalence of 31.4% for hypertension [10], a notable increase from the previous reports [6–8]. Looking at the existing burden of disease, it is imperative for healthcare professionals to be aware of the risk factor associated with high blood pressure for this population. Unless steps are taken to increase awareness among the Saudi population, this trend of increasing hypertension-related mortality is most likely to persist. The Seventh Report of the Joint National Committee on Prevention, Detection, Evaluation, and Treatment of High Blood Pressure (JNC 7) presented the novel class of pre-hypertension, defined as systolic BP of 120 to 139 mm Hg and/or diastolic BP of 80 to 89 mm Hg [11, 12].

El Bcheraoui and colleagues observed that older age, male gender, obesity, and a previous diagnosis with diabetes are all risk factors for hypertension [13]. Hypertension was poorly controlled among Saudis and the majority were unaware of their condition [13]. The reports on high blood pressure and other related morbidities’ reports for the Saudi population in general [9], have led to the development and implementation of guidelines for treatment [7, 8]. Furthermore, no studies have evaluated subsets from the Saudi population such as the population of Alkharj, and no specific information about the prevalence of hypertension and its associated cardioembolic factors for this population exist. This article estimated the prevalence and cardioembolic risk factors of hypertension and prehypertension among this group.

## Methods

### Study design

A population based cross sectional study was conducted between January 2016 and June 2016 to determine the hypertension and pre-hypertension prevalence and its associated cardioembolic risk factors in Al Kharj, Saudi Arabia.

### Settings

The study was conducted in Al Kharj, a city located in Al Kharj Governorate in central Saudi Arabia which is 77 km south of Riyadh, with a population of more than 376,000. It has mixed urban (military and civilian), rural, and adjacent nomadic communities.

### Sample size/sampling

Data was collected from January 2016 to June 2016 with a total sample size of 1200, including a response rate of 85%. Complete data was available for 1019 individuals where 381 were males and 638 were females. A multi-stage sampling method was used. Surveys of the same study population had also been conducted previously [14]. List of all clusters were made, and a random number of clusters was drawn by the investigators to be included in the study. Samples from 21 governmental and 11 private institutes were selected through a cluster sampling technique. The total population of these institutions were divided into groups called clusters after acquiring a list of participants in each institute nominated. Samples of the respondent were then selected using simple random sampling from each of the group (cluster). Eligible study subjects included all Saudi citizens 18 years and older who showed willingness to participate in the study.

### Instruments

A structured self- administered 20 items Arabic questionnaire was formulated. It was based on the review of the previously published literature. The questionnaire evaluated the socio-demographic characteristics of the participants including age, education, and marital status. In addition, information about smoking status and chronic medical illnesses were collected. Participants were examined for physical parameters including blood pressure, weight, height, waist circumference, and pulse rate. Blood samples from all participants were examined for the following cardiometabolic measurements: Cholesterol, HDL and HbA1c.

### Measures

The anthropometric measurements such as body weight, waist circumference, height, blood pressure, and pulse rate were collected by well-trained nurses.

Blood pressure (BP) was measured using stethoscope and a mercury sphygmomanometer (adult size). The JNC 8 guidelines were followed as a measurement criterion [15, 16]. The BP of the participants was measured while the individual was sitting on a chair. The BP was measured from the right arm. It was ensured that the individual took rest for at least 5 min before taking the measurement. A specific point at which the initial Korotkoff sound was heard, was noted as SBP, whereas the DBP was measured at the point on which this sound disappeared.

Height and body weight were also measured with individuals wearing light clothing or standing without shoes. Body weight was measured through a digital weighing scale. Prior to the measurement, the scale was calibrated to the zero level and was also verified for repeatability of the readings. Height was also measured, while the participants were in an upright position, by using a Health O Meter Digital Scale (made in USA) which reads to the adjoining 100 g. It was used as the weighing scale. A flexible tape meter was used to measure the waist and hip circumferences equally, at the level directly above the iliac crest and at the maximum circumference of the hip, respectively. Waist circumference (WC) was used as surrogate for abdominal obesity, and a WC value less than 102 cm was considered normal WC in men, while a value less than 88 cm was considered normal in women. Body mass index (BMI) was computed as weight in kilograms divided by height in meters squared (kg/m^2^).

### Operational definition

Prehypertension was defined as having either a SBP of 120 to 139 mmHg and/or DBP of 80 to 89 mmHg -- according to JNC7, in individuals who were not on antihypertensive medication [11, 12]. Hypertension was defined as presence of persistently elevated BP or a history of treatment with antihypertensive agents [16]. Overweight status is defined as a BMI of 25 kg/m^2^ or more, and obesity as having a BMI of 30 kg/m^2^ or more [14].

### Data analysis

The data collected was cleaned and entered into a PC to be examined by using SPSS version 24.0 for Windows. Categorical variables such as gender, educational level, and age groups were summarized and reported in terms of frequency distribution. A chi-square test was used to examine the association between different categorical variables whereas t-test or ANOVA were used for continuous variables. Multivariate and logistic regression analysis were performed to comprehend the relationship between independent and dependent variables. *P*-values of <0.05 were considered statistically significant.

### Ethics approval

The study ethics approval was acquired from the local Institutional Review Board (i.e., Committee of Scientific Research and Publication). Both verbal and written consent was attained from all respondents after an inclusive elucidation of the procedure and purpose of the study was given. The identity of the participants as well as their personal information was kept confidential. Participants incidentally reporting with life-threatening conditions during the period of this survey were immediately referred to the hospitals, and those individuals with new incidence of hypertension were also directed to begin a follow-up treatment at the nearby health facility or a hospital.

## Results

Of the total 1019 participants, 381 (37.4%) were males, while, 638 (62.6%) were females. The study population age ranged from 18 to 67 years. There were 71.9% participants aged 18–29 years, 18.3% aged 30–39 years, 7.3% aged 40–49 years, and only 2.6% aged 50–67 years. The prevalence of prehypertension was 66.1, 48.1 and 54.9% in male, female and all subjects, respectively. The prevalence of hypertension was 6.0, 4.2 and 4.9% in male, female and all subjects, respectively.

### Univariate analysis

Based on the status of their blood pressure, eligible study participants in the Al-Kharj study (*n* = 1019) were classified into three groups: normotensive (*n* = 743), pre-hypertensive (*n* = 559), and hypertensive (*n* = 50). Table 1 illustrated some descriptive univariate data for “systolic blood pressure” (SBP) and “diastolic blood pressure” (DBP) for each of the three-blood pressure (BP) groups. Descriptive statistics include the mean, median, standard deviation (SD), interquartile range, minimum and maximum data.

**Table 1:** Description of SBP and DBP of normotensive, pre-hypertensive, and hypertensive individuals in the Al Kharj study (*n* = 1019)

Variable	Statistics	Normotensive (*n* = 743)	Pre-hypertensive (*n* = 559)	Hypertensive (*n* = 50)	Total (*n* = 1019)
SBP (mmHg)	Mean	117.98	129.55	153.52	123.12
	SD	11.08	8.47	13.20	14.78
	Median	118.00	129.00	151	122.00
	Interquartile range	76	59	77	148
	Minimum	80	102	140	69
	Maximum	156	161	217	217
DBP (mmHg)	Mean	71.78	78.80	99.32	75.81
	SD	6.5	7.35	10.87	10.28
	Median	72	80	97	75
	Interquartile range	57	58	53	103
	Minimum	40	45	90	40
	Maximum	97	103	143	143

*SBP* systolic blood pressure, *DBP* diastolic blood pressure

The prevalence of hypertension seemed to increase with the increasing age. Table 2 showed that there is a linear proportional significant increase in hypertension status for each of the following three age groups: *30–39 years* (16.2% for normotensive people; 19.5% for pre-hypertensive people; and 24% for hypertensives) where *P* < 0.001; *40–49 years* (5.2% for normotensives; 9.3% for pre-hypertensives; and 16.0% for hypertensives) where *P* < 0.001; and most notably for the oldest age group; namely, the *50–67 years* (1.9% for normotensives; 3% for pre-hypertensives; and 8% for hypertensives) where *P* < 0.001. With respect to gender, there were more females than males in each of the BP groups. For example, there were 54.9% pre-hypertensive females vs. 45.1% males. Likewise, the proportion of females in the hypertensive category (54%) was higher than their male counterparts (46%). Being single (not married) was associated with a higher risk of pre-hypertension (62.8%) and hypertension (52%), in comparison with those married with pre-hypertension (37.2%) and those married with hypertension (48%).

**Table 2:** Sociodemographic profile among normotensive, pre-hypertensive, and hypertensive individuals in the Al Kharj Study (*n* = 1019)

Variables	Normotensive (*n* = 743)	Prehypertensive (*n* = 559)	Hypertensive (*n* = 50)	Total (*n* = 1019)
	n (%)	n (%)	n (%)	n (%)
Age group				
18–29 years	570 (76.7)	381 (68.2)	26 (52.0)	733 (71.9)
30–39 years	120 (16.2)	109 (19.5)	12 (24.0)	186 (18.3)
40–49 years	39 (5.2)	52 (9.3)	8 (16.0)	74 (7.3)
50–67 years	14 (1.9)	17 (3.0)	4 (8.0)	26 (2.6)
Gender				
Male	248 (33.4)	252 (45.1)	23 (46.0)	381 (37.4)
Female	495 (66.6)	307 (54.9)	27 (54.0)	638 (62.6)
Marital status				
Not married	504 (67.8)	351 (62.8)	26 (52.0)	662 (65.0)
Married	239 (32.2)	208 (37.2)	24 (48.0)	357 (35.0)
Education level				
Primary	14 (1.9)	12 (2.1)	5 (10.0)	22 (2.2)
Secondary	92 (12.4)	72 (12.9)	10 (20.0)	131 (12.9)
Intermediate	21 (2.8)	20 (3.6)	2 (4.0)	30 (2.9)
University	594 (79.9)	432 (77.3)	32 (64.0)	800 (78.7)
Postgraduate	22 (3.0)	23 (4.1)	1 (2.0)	36 (3.5)

Table 3 illustrates categorization of Body mass index (BMI) into four standard groups: non-obese (< 25 kg/m^2^), overweight (25–29.9 kg/m^2^), class I obese (30–34.9 kg/m^2^), and class II/III obese (≥ 35 kg/m^2^). It was found that the proportion of individuals who were considered non-obese and/or overweight were higher in the normotensive BP group compared with their counterparts in the prehypertensive or hypertensive groups. It was notable that there was a linear proportional increase in the prevalence of hypertension for each of Class I obese (19.2% for prehypertensive; and 36% for hypertensives) and Class II/III obese (15.2% for prehypertensive; and 26% for hypertensives). All these differences were statistically significant, where *P* < 0.001. Regarding cardiometabolic risk factors, we were able to assess serum cholesterol levels. Hypertensive individuals showed significantly higher levels of cholesterol (22%) than the prehypertensive (13.8%) and normotensive (10.6%) individuals. Similarly, low levels of HDL (HDL <  1) were observed in hypertensive (34%), and prehypertensive individuals (21.5%) more so than normotensives (16.7%). This indicates that low levels of the good cholesterol (HDL) is a risk factor for hypertension.

**Table 3:** Comparison among normotensive, pre-hypertensive, and hypertensive individuals according to BMI and other cardiometabolic variables in the Al Kharj Study (*n* = 1019)

Variables	Normotensive (*n* = 743)	Prehypertensive (*n* = 559)	Hypertensive (*n* = 50)	Total (*n* = 1019)
	n (%)	n (%)	n (%)	n (%)
BMI Group				
Non-obese (< 25 kg/m^2^)	374 (50.3)	217 (38.9)	10 (20.0)	465 (45.7)
Overweight (25–29.9 kg/m^2^)	215 (28.9)	149 (26.7)	9 (18.0)	272 (26.7)
Class I obese (30–34.9 kg/m^2^)	104 (14.0)	107 (19.2)	18 (36.0)	169 (16.6)
Class II & III obese (≥ 35 kg/m^2^)	50 (6.7)	85 (15.2)	13 (26.0)	112 (11.0)
Cardiometabolic variables				
Cholesterol (≥ 5.6)	79 (10.6)	77 (13.8)	11 (22)	127 (0.12)
HDL (< 1)	124 (16.7)	120 (21.5)	17 (34.0)	187 (18.1)
HBA1c (< 5.7%)	568 (76.4)	391 (69.9)	28 (56.0)	743 (72.9)
5.7–6.4%	153 (20.6)	141 (25.2)	15 (30.0)	231 (22.7)
≥ 6.5%	22 (3.0)	27 (4.8)	7 (14.0)	45 (4.4)

High levels of HbA1c (5.7–6.4%) were significantly higher in both hypertensive (30%) and prehypertensive (25.2%) than in normotensive (20.6%) persons. Furthermore, elevated HbA1c levels (≥ 6.5%) were significantly higher in hypertensive people (14%) than in either the prehypertensive (4.8%) or the normotensive (3%) individuals, where *P* < 0.001.

#### Multivariate logistic regression analysis

First, we conducted a multiple logistic regression analysis to determine the risk of *pre-hypertension*, as the dependent variable using the entire sample (*n* = 1019) in Table 4. After adjusting for age and other sociodemographic variables (marital status, and education attainment level), we found that the risk of pre-hypertension was significantly lower in females (compared to males); Odds ratio (OR) = 0.48 [95% C.I. = 0.32–0.71], *P* < 0.0001. The risk of pre-hypertension had a tendency to increase with obesity status, in that the overweight, class I obese, and class II/III obese statuses were all associated with significantly increased risk of pre-hypertension (Table 4). Other variables included in the logistic regression model were not statistically significant - these included serum cholesterol, diabetes status, and smoking status.

**Table 4:** Logistic regression model to predict prehypertension from sociodemographic and other variables adjusting for age in the Al Kharj Study (*n* = 1019)

Variable	B	S.E. of B	Sig.	Exp (B)/ Odds ratio	95% C.I. for Odds ratio	
					Lower	Upper
Age in years	−.015	.013	.273	.986	.960	1.012
Gender						
Female	−.742	.205	.000	.476	.319	.712
Marital status						
Married	−.342	.190	.072	.710	.489	1.031
Education level			.144			
Primary	.533	.604	.377	1.704	.522	5.568
Secondary	.485	.413	.240	1.624	.723	3.648
Intermediate	−.006	.551	.992	.994	.338	2.926
University or postgraduate	−.062	.383	.870	.939	.444	1.988
BMI			.000			
Overweight/BMI = 25 kg/m^2^	1.216	.256	.000	3.374	2.045	5.568
Class I obese/BMI = 30–34.9 kg/m^2^	.940	.260	.000	2.560	1.537	4.264
Class II/III obese/BMI ≥ 35 kg/m^2^	.593	.279	.033	1.810	1.048	3.126
Cholesterol	−.078	.083	.348	.925	.785	1.089
Diabetes	−.307	.359	.393	.736	.364	1.488
Smoking Status			.922			
Never Smoked/Ex-Smoker	−.048	.254	.850	.953	.579	1.569
Current Smoker	.108	.443	.807	1.114	.467	2.657
Constant	.445	.890	.617	1.561		

Second, we conducted another multiple regression analysis for prediction of risk of *hypertension* again using the entire sample (*n* = 1019) in Table 5. We found that increasing BMI was associated with a significantly increased risk of hypertension. Being overweight (25–29.9 kg/m^2^) was associated with the highest risk of hypertension (OR = 4.98 [95% C.I. = 1.98–12.52], *P* = 0.001). Further, people who were classified as class I obese (30–34.9 kg/m^2^) had 3.5 times the risk of hypertension compared with the non-obese (< 25 kg/m^2^) group (OR = 3.49 [95% C.I. = 1.42–8.63], *P* = 0.007).

**Table 5:** Logistic regression model to predict hypertension from sociodemographic and other variables adjusting for age in the Al Kharj study (*n* = 1019)

Variable	B	S.E. of B	Sig.	Exp (B)/ Odds ratio	95% C.I. for Odds ratio	
					Lower	Upper
Age in years	−.008	.023	.722	.992	.948	1.038
Gender						
Female gender	−.380	.443	.390	.684	.287	1.628
Marital status						
Being Married	.051	.403	.899	1.052	.478	2.318
Education level			.222			
Primary	−1.954	1.207	.106	.142	.013	1.510
Secondary	−1.305	1.086	.229	.271	.032	2.277
Intermediate	−.996	1.295	.442	.369	.029	4.671
University or postgraduate	−.627	1.061	.554	.534	.067	4.274
BMI			.000			
Overweight/BMI = 25 kg/m^2^	1.604	.471	.001	4.975	1.977	12.522
Class I obese/BMI = 30–34.9 kg/m^2^	1.251	.461	.007	3.494	1.415	8.629
Class II/III obese/BMI ≥ 35 kg/m^2^	.080	.400	.841	1.084	.495	2.374
Cholesterol	−.063	.172	.714	.939	.671	1.315
Chronic disease Type						
Diabetes (yes)	.516	.532	.332	1.675	.591	4.746
Smoking Status			.981			
Never Smoked/Ex-Smoker	−.094	.525	.858	.910	.325	2.546
Current Smoker	−.011	.885	.990	.989	.175	5.605
Constant	2.736	1.832	.135	15.422		

#### Gender-based logistic regression analysis

We conducted four other logistic regression analysis models to using gender as a stratification variable (1 = male; 2 = female) in SPSS 24.0 for Windows. *First*, we ran two models to determine the risk of pre-hypertension in males and females. *Second*, we ran two other models to determine the risk of hypertension in males and females. In one logistic regression model, we found that males in the lowest education attainment level (primary level) had a significantly increased risk of *pre-hypertension* (OR = 6.56 [95% C.I. = 1.27–33.85], *P* = 0.003). No significant prediction of risk of pre-hypertension/hypertension pertaining to either the lowest education level was found among females in the other logistic regression analyses. Our results are consistent with the Whitehall II studies [17, 18].

## Discussion

High blood pressure is a condition of major concern because of its role in the causation of vascular complications, coronary heart disease (CHD), and stroke [5]. The overall burden of hypertension–associated diseases is increasing possibly due to an epidemiological transition, an aging population, urbanization and an increase in age-specific rates of many chronic conditions [9]. The present study highlights major risk factors for cardiovascular diseases in Saudi Arabia among the general, healthy population of Al Kharj. The overall estimated prevalence of hypertension presented in this report is significantly lower when compared to previous nationally conducted studies [10] except for the older age group that exhibited a compatible number [10, 13, 19]. The overall proportion of pre-hypertension seems to be alarmingly higher than previous reports among all age groups [9, 20]. The evidence that hypertension and pre-hypertension increase with age, as noted in this study, is a well-documented fact [21]. Similarly, very few studies in the literature noted a higher prevalence of hypertension in women than men [22], while the majority suggested higher prevalence in men [8, 23]. This is an indication that the population of Al-Kharj being studied in this report has unique features, and it is likely that genetic composition, physiological, and environmental characteristics all have contributed to the variation in the prevalence of hypertension between men and women in this semi-urban area. Hypertension was also higher among the Class I and Class II/III BMI groups (Table 3), which is compatible with previous reports in the country [8, 10, 24]. Central obesity is well known to be significantly associated with hypertension [21, 25]. Moreover, subjects who reported being single presented with higher blood pressure than the married subjects. A study by Lipowicz et al. reported that being single was significantly associated with being in higher blood pressure groups [26]. Furthermore, biochemical markers such as high levels of HBA1c, high levels of serum cholesterol, and low levels of HDL were also observed to be significantly associated with high blood pressure, as reported in other studies [27, 28]. High levels of HbA1c in this study were significantly higher in both the hypertensive and pre-hypertensive participants as compared to normotensive participants. This finding emphasizes on the importance of diabetes screening once hypertension is diagnosed and vice versa.

Multivariate logistic regression analysis identified female gender and obesity as major risk factors for hypertension and pre-hypertension in this semi-urban population, while controlling for age and other sociodemographic characteristics (marital status, and education attainment level). Though some previous studies conducted in the last few years suggest that male gender is a risk factor in Saudi Arabia, the picture in a region like Al-Kharj with a semi-urban agricultural environment is not similar. The disparate results from diverse locations in Saudi Arabia indicate that different regions of Saudi Arabia are in different stages of epidemiological transition, and there is a growing susceptibility for cardiovascular risk factors among women. In multivariate analysis among males only, education was an independent risk factor of pre-hypertension, as reported in other studies [13, 29]. Wang and colleagues found that BP was associated inversely with the level of education regardless of all other risk factors [2]. Educated people are most expected to be aware of their health conditions and of taking preventive measures to avoid complications.

Our study revealed hypertension and prehypertension to be prevalent particularly among males. A similar national study conducted in Saudi Arabia revealed that hypertension was more prevalent among males (18.7%) compared to females (14.0%). The highest prevalence of hypertension was reported in the Eastern province of Saudi Arabia [30]. “While other studies indicated that prehypertension was documented to be more prevalent among males than females, regardless of age [31–34].”

This study has few limitations. First, with the cross-sectional design it is challenging to generalize the results to the whole population of Saudi Arabia. The collected data was restricted to the active population; thus, results from this study are not generalizable to persons who were non-active population (such as retired, or workless people) and that if they were included, the figures would likely to be higher. Our study was conducted in Al-Kharj and not in other regions, generalizing our results to Saudi Arabia might be of question, however, the characteristics of the population of Al Kharj is comparable to that of all Saudi Arabia, which is mixed between urban and rural population. A second limitation is that we were unable to assess family history of hypertension, dietary effects, and levels of physical activity in the current population; and how these factors may have impacted hypertension. This issue is due to a lack of valid and reliable methods to quantify these parameters in this population. We do not expect that levels of physical activity would have influenced our results because most of our population were overweight or obese.

Despite these limitations, this study is important and highlights the situation of hypertension in an agricultural community undergoing a major transition into the era of chronic diseases.

## Conclusion

Overall, the results of the current study reported hypertension and prehypertension to be prevalent particularly among adult males. We were able to identify that overweight and obesity were associated with both prehypertensive and hypertensive states. Our data also provide evidence that lower education attainment was predominant among adult males in Al-Kharj region. Community awareness campaigns directed towards increasing individual’s knowledge of risk factors for prehypertension and hypertension are warranted. Furthermore, well-designed prospective studies are needed in future to assess the association of dietary patterns (effects) and levels of physical activity to the risk of prehypertension and hypertension statuses, as outcomes.

## Abbreviations

BMI: Body mass index
BP: Blood pressure
CHD: Coronary heart disease
CI: Confidence interval
DBP: Diastolic blood pressure
OR: Odds ratio
SBP: Systolic blood pressure
WC: Waist circumference
